# Nutritional, Techno-Functional and Structural Properties of Black Soldier Fly (*Hermetia illucens*) Larvae Flours and Protein Concentrates

**DOI:** 10.3390/foods11050724

**Published:** 2022-02-28

**Authors:** Vusi Vincent Mshayisa, Jessy Van Wyk, Bongisiwe Zozo

**Affiliations:** 1 Department of Food Science and Technology, Cape Peninsula University of Technology, Bellville 7535, South Africa; vanwykj@cput.ac.za; 2 Department of Chemistry, Cape Peninsula University of Technology, Bellville 7535, South Africa; 214050645@mycput.ac.za

**Keywords:** functional properties, black soldier fly larvae, protein extraction, foaming, insect protein, novel food ingredients

## Abstract

Due to their protein content and balanced amino acid profile, edible insects have been described as an excellent alternative protein source to combat malnutrition. As the global population continues to grow, edible insects such as the black soldier fly larvae (BSFL) may contribute to food security. The effect of different protein extraction methods, i.e., alkaline solution and acid precipitation (BSFL-PC1) and extraction with an alkali (BSFL-PC2), on the nutritional, techno-functional, and structural properties of BSFL flours and protein concentrates were studied. The highest protein content (73.35%) was obtained under alkaline and acid precipitation extraction (BSFL-PC1). The sum of essential amino acids significantly increased (*p* < 0.05) from 24.98% to 38.20% due to the defatting process during extraction. Protein solubility was significantly higher in protein concentrates (85–97%) than flours (30–35%) at pH 2. The emulsion capacity (EC) was significantly higher (*p* < 0.05) in the protein concentrates (BSFL-PC1 and BSFL-PC2) compared to the freeze-dried and defatted BSFL flours, while the emulsion stability (ES) was significantly (*p* < 0.05) higher in BSFL-PC1 (100%) compared with BSFL-PC2 (49.8%). No significant differences (*p* > 0.05) were observed in foaming stability (FS) between freeze-dried and defatted BSFL flours. Fourier transform infrared spectroscopy (FT-IR) analysis revealed distinct structural differences between BSFL flours and protein concentrates. This was supported by surface morphology through scanning electron microscopy (SEM) images, which showed that the protein extraction method influenced the structural properties of the protein concentrates. Therefore, based on the nutritional and techno-functional properties, BSFL flour fractions and protein concentrates show promise as novel functional ingredients for use in food applications.

## 1. Introduction

The United Nations (UN) has predicted that the world population will increase from seven to nine billion people by the year 2030 [1], and with this increase, about 60% of people are expected to migrate and live in cities [2]. Animal protein is expensive and is becoming beyond the reach of many people, especially in developing countries. To feed this growing population, a paradigm shift towards producing sustainable and cost-effective food products is required now more than ever before. Entomophagy, or the consumption of insects, has been practised by humankind on every continent in the world throughout history and continues today. However, the advancement of the scientific impetus for large-scale insect rearing, production, and utilisation began in 1975 with the call by Meyer-Rochow [3]. Over 2000 species have been deemed edible since 2012 in 116 countries, and there has been an increase in industrial insect rearing companies in recent years [4,5]. Large industrial-scale insect farming companies include AgriProtein in South Africa, Ynsect in France, Enviroflight in Ohio, USA, and HaoCheng Mealworms Inc. in China [6]. Edible insects have recently been suggested as a food source that could address economic, environmental, and health concerns as the population expands. Due to the emerging demand in edible insect consumption, the industrial rearing of edible insects such as the black soldier fly larvae (*Hermetia illucens*, hereinafter referred to as BSFL) has been expanding due to its stable supply, cost-effectiveness, and hygienic production. Currently, edible insects are primarily marketed as whole insects, ground pastes or flours, protein powders, and oil fractions which can further be used as ingredients in food applications [2,7,8]. Edible insects are a good source of minerals and vitamins and fat, and, most importantly, protein. According to La Barbera et al. [9], consumers may be willing to consume insects when they are added as an ingredient in an unrecognisable form. The food industry’s further processing of insects as an alternative protein source and acceptance depends mainly on their ability to fulfil tailored techno-functional properties required in food systems. The potential use of an insect ingredient as a techno-functional food ingredient is dependent on chemical, physical, techno-functional, and structural properties [10]. 

An ingredient’s techno-functionality has been described as any food property, excluding its nutritional value, affecting its utilisation [11,12]. Emulsification, solubility, water and oil binding, foam capacity and stability, gelation, and viscosity are among the techno-functional properties vital in food processing. Yi et al. [13] studied five different acid-extracted insect proteins and reported that these exhibited poor foaming and gelling. Protein-enriched fractions of *Schistocerca gregaria* (*S. gregaria)* and *Apis mellifera* (*A. mellifera)* were reported to have a higher foam stability after alkaline and sonication extraction, respectively. Moreover, Akpossan et al. [14] demonstrated that *Imbrasia oyemensis* (*I. oyemensis*) protein fractions from full fat and defatted flours possessed poor solubility due to their isoelectric point. However, both studied flours exhibited good water absorption and emulsification characteristics. According to Omotoso [15], the dried powders of *Cirina forda (C. forda)* possessed good emulsion and solubility properties. Kim et al. [7] evaluated the effects of added defatted *Tenebrio molitor* (*T. molitor*) and *Bombyx mori (B. mori)* in sausages. Including edible insect flours increased cooking yield and firmness in the emulsion-based sausages. For the successful application of insect-derived ingredients in food formulations, it is essential to understand their nutritional and techno-functional properties and how processing affects them.

To date, an in-depth analysis of the nutritional value of commercially available *H. illucens* available in South Africa has not been reported. For the possible use of *H. illucens* flours and protein concentrates as foodstuffs, information on the nutritional value and techno-functional properties are extremely essential. Therefore, the aim of this study was to determine the effect of different extraction methods on the nutritional, techno-functional, and structural properties of BSFL flour and protein concentrates in order to identify new protein sources for human nutrition.

## 2. Materials and Methods

### 2.1. Chemicals

If not stated otherwise, all chemicals were purchased from either Merck (Modderfontein, South Africa) or Sigma-Aldrich (Aston Manor, South Africa). All the chemicals used in this study were of analytical grade, and chemical reagents were prepared according to standard analytical laboratory procedures. Ultrapure water purified with a Milli-Q water purification system was used throughout the experiments conducted (Millipore, Microsep, Cape Town, South Africa). The study was approved by the faculty of applied sciences ethics committee (Ref: 208176519/01/20220 date: 4 February 2020).

### 2.2. Edible Insects Flour Preparation

**Figure 1:**
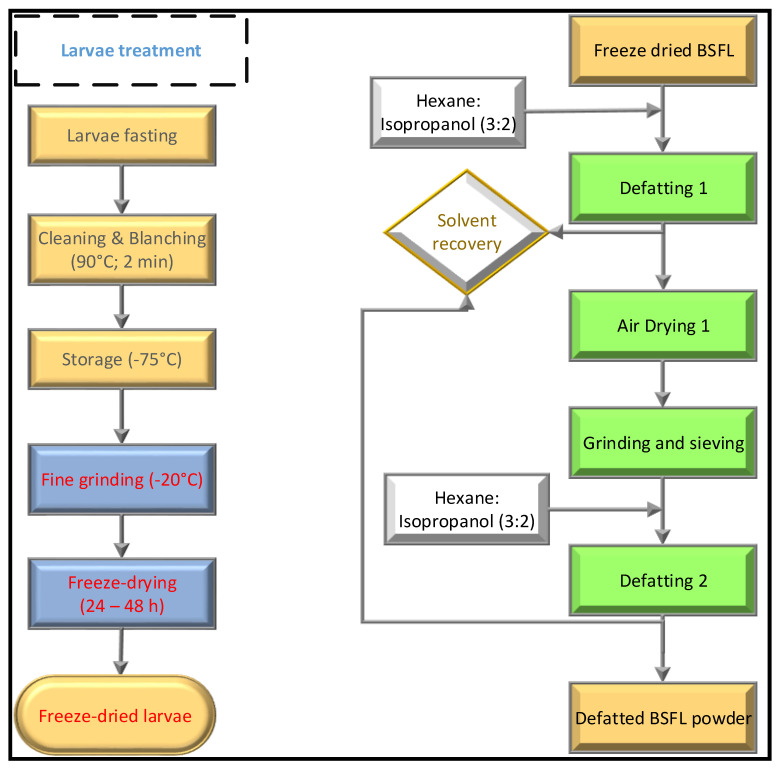
BSFL pre-treatment and defatting.

Previously fasted *Hermetia illucens* (black soldier fly—BSF) in the larval stage were obtained from AgriProtein, Cape Town, South Africa. They were immediately blanched in boiling water for two minutes to prevent browning and washed with cold water (Figure 1). To obtain a paste, the clean larvae were frozen at −75 °C in a blast freezer prior to grinding in a laboratory blender. This paste was then freeze-dried (Genesis, Virtis, New York, NY, USA) to obtain a stable powder. Some of the freeze-dried (BSFL-FD) powder was ground in a laboratory blender (Kenwood, Titanium, South Africa) and was passed through a 40-mesh US Standard sieve (Endecotts, London, UK) to separate the integument and stored at −20 °C until further analysis. The sieved BSFL flour was defatted using hexane and isopropanol (3:2) mixture. One part of the insect flour and five parts of defatting solvent were stirred in a magnetic stirrer for six hours. After the solids were sedimented, the solvent-fat mixture was decanted, and the procedure was repeated twice. Residual hexane was removed by evaporation overnight in a fume hood, and the defatted flour (BSFL-DF) was stored at −20 °C until further analysis.

### 2.3. Preparation of Insect Proteins

**Figure 2:**
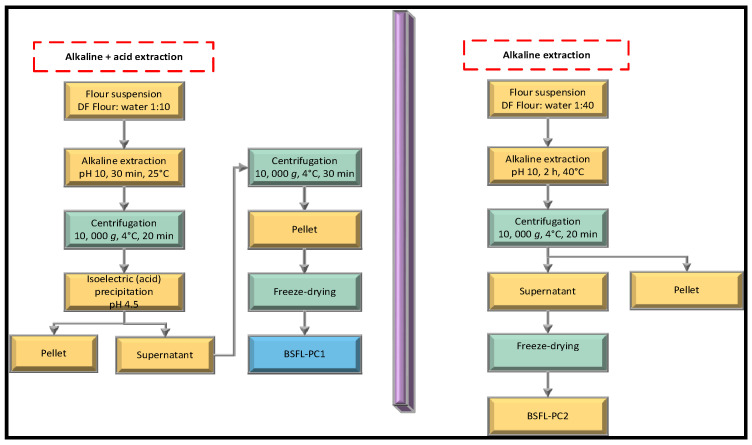
Alkaline and acid precipitation extraction (BSFL-PC1) and alkaline extraction (BSFL-PC2) of protein concentrates.

This study used two chemical techniques, alkaline solution and isoelectric precipitation (IEP) and alkaline extraction, to extract protein concentrates from defatted BSFL flour (Figure 2).

#### 2.3.1. Alkaline and Isoelectric Precipitation

The BSFL protein concentrate (BSFL-PC1) was prepared as previously described [16]. Briefly, after defatting, BSFL flour (BSFL-DF) was mixed with Milli-Q water at a ratio of 1:10 (*w*/*w*), and the pH of the mixture was adjusted to pH 10 using 1 M NaOH solution (Figure 2). With the aid of a magnetic stirrer, the slurry was stirred for 30 min (25 °C ± 1.0) on a laboratory mixer at a rate intended to prevent the formation of a vortex. Subsequently, the slurry was centrifuged at 10,000× *g* for 20 min (4 °C). The pH of the supernatant was then adjusted to 4.5 with 1 M HCl, and the suspension was left at 4 °C overnight to allow protein precipitation. Centrifugation at 10,000× *g* for 30 min (4 °C) was used to recover the precipitated soluble proteins. The BSFL protein concentrate (BSFL-PC1) was then freeze-dried (0.094 mBar, −74.5 °C, 48 h, Genesis, Virtis, New York, NY, USA) and stored in vacuum bags at −20 °C.

#### 2.3.2. Alkaline Extraction

Following the method of Azagoh et al. [10], with some modifications, BSFL-PC2 was extracted by mixing defatted BSFL flour with Milli-Q water at a ratio of 1:40 (*w*/*w*), and the pH of the mixture was adjusted initially to pH 10 using 1 M N NaOH solution (Figure 2). Subsequently, the slurry was stirred at a rate designed to prevent the formation of a vortex for 2 h at 40 °C. The pH was monitored intermittently and maintained at 10 throughout the stirring period. The mixture was centrifuged at 10,000× *g* for 30 min (4 °C). The BSFL protein concentrate (BSFL-PC2) supernatants were freeze-dried under similar experimental conditions with BSFL-PC1. 

### 2.4. Proximate Composition Analysis

The proximate composition, i.e., the moisture (925.10), crude protein (920.87), crude fat (932.06), and ash content (923.03) of the insect flour and protein concentrates, was determined following standard methods recommended by the Association of Official Analytical Chemists [17]. Moisture percentage was calculated by drying the sample in an oven at 100 °C for two hours. The dried sample was placed into a desiccator, allowed to cool, and then re-weighed. The process was repeated until a constant weight was obtained. Crude protein content was analysed by the high-temperature combustion process according to the Dumas combustion method (TruSpec-N Leco, St. Joseph, MI, USA) using a protein-to-nitrogen conversion factor of 5.60 as recommended by Janssen et al. [18]. EDTA was used as a standard. Crude fat was determined by drying fats after extraction in a Soxhlet assembly using diethyl ether. The ash percentage was calculated by combusting the samples in a silica crucible placed in a muffle furnace at 550 °C. The percentage of carbohydrates on a dry basis was determined by subtracting all of the components (moisture, crude protein, crude lipid, and ash) from 100.

### 2.5. Amino Acid Analysis

Five hundred milligrams of each insect flour and protein concentrate were hydrolysed with 6 mL of 6 N HCl at 110 °C for 23 h. Then the internal standard (7.5 mL of 5 mM norleucine in water) was added. The hydrolysed samples were analysed by High-Performance Liquid Chromatography with a fluorescence detector (HPLC/FLD, Waters Alliance 2695, Agilent technologies, Chemetrix, Midrand, South Africa) after derivatisation with 6-aminoquinolyl-N-hydro-xysuccinimidyl carbamate (AQC). The amino acid profiles of insect flour and protein concentrates were compared with data in the literature on egg white and cow’s milk proteins used for human consumption. The FAO/WHO method was used to calculate the amino acid score (AAS) as shown below:

$$\mathrm{AAS}=\frac{\mathrm{mg}\;\mathrm{of}\;\mathrm{AA}\;\mathrm{in}\;1\;g\;\mathrm{of}\;\mathrm{test}\;\mathrm{protein}}{\mathrm{mg}\;\mathrm{of}\;\mathrm{AA}\;\mathrm{in}\;1\;g\;\mathrm{of}\;\mathrm{the}\;\mathrm{FAO}/\mathrm{WHO}\;\mathrm{reference}\;\mathrm{pattern}}\times 100$$

### 2.6. Bulk Density

Bulk density was measured as a ratio of mass to volume. A graduated cylinder, previously tared, was gently filled up to the ten mL mark with BSFL flour or protein. The sample was then packed by gently tapping the cylinder on the bench-top from a height of five cm until there was no further diminution of the sample level, and the volume was noted. The weight of the filled cylinder was taken, and the bulk density was calculated as the weight of sample per unit volume (g/mL).

### 2.7. Colour

Colour was determined by the method as described by Diedericks and Jideani [19] and Bußler et al. [20], with minor modifications. The colour of the insect flours and protein concentrates was measured with a spectrophotometer (Konica Minolta Sensing Americas Inc., Tokyo, Japan) using the CIEL* a* b* colour space system. The instrument was calibrated by using the white calibration plate followed by zero calibration. Powdered samples were placed evenly in the provided cuvette (diameter 30 mm), covering the bottom of the dish, to allow for reflectance measurement. Measurements for each sample were performed in triplicate at 3 different positions in the samples (one reading = average of 3 readings per rotated position), with the results recorded in L* (lightness), a* (+a* = red and −a* = green), b* (+b* = yellow and −b* = blue). The colour change (ΔE) was calculated, whereas the indices 0 and s indicate measured values of unprocessed (larvae) and processed insects (flour fractions), respectively.

$$\Delta E=\sqrt{{\left(L_{0}-L_{s}\right)}^{2}+{\left(a_{0}-a_{s}\right)}^{2}+{\left(b_{0}-b_{s}\right)}^{2}}$$

where: L—is L* (lightness), a—a* (difference in green and red), b—b* (difference in yellow and blue), o—indicates measured values of BSFL-FD, s—indicates measured values of processed flour or protein concentrates.

### 2.8. Determination of Techno-Functional Properties

#### 2.8.1. Water and Oil Binding Capacity

The water-binding capacity (WBC) was determined according to Purschke et al. [21], with slight modifications. Briefly, 0.5 g insect flour or protein concentrate powder was mixed with 2.5 mL Milli-Q water at an ambient temperature (25 °C), vortexed for 1 min (Vortex-Genie 2, Scientific Industry Inc., Bohemia, NY, USA), and centrifuged for 2 min at 3330× *g*. The non-bound water was decanted, and the tube was placed upside down on a Whatman No 1 filter paper for one hour and re-weighed. WBC was calculated as:

$$\mathrm{WBC}=\frac{W_{b}-W_{a}}{W_{a,\mathrm{DM}}}$$

where: W_a_ is the initial weight, W_b_ is the final weight, and W_a,DM_ is the initial weight of the sample based on dry matter. The oil binding capacity (OBC) was analysed using commercial sunflower oil instead of Milli-Q water. Except for the vortexing step (2 min), the experimental procedure was identical to the WBC assay, and OBC was calculated similarly.

#### 2.8.2. Emulsion Capacity and Stability

Emulsifying properties were determined based on the method of Coelho and Salas-Mellado [22] and Zielińska et al. [23] with minor revisions. Briefly, the edible insect sample was dispersed in distilled water (5% *w*/*v*) and centrifuged (Thermo Electron Corporation Jouan MR1812, Waltham, MA USA) at 9000× *g* speed for 15 min. The supernatant was mixed with sunflower oil (1:1 *v*/*v*) and homogenised (Polytron PT 2500 E, Thermofisher, Cape Town, South Africa) at 18,000 rpm for 1 min. Aliquots (15 mL) of the emulsion samples were centrifuged at 3000× *g* for five min, and the volume of the individual layers was read using a 50 mL scaled tube. The emulsifying activity was calculated as:

$$\mathrm{Emulsion}\;\mathrm{capacity}\;(\mathrm{EC})=\frac{V_{\mathrm{el}}}{V}\times 100$$

The emulsion was heated in a water bath (80 °C for 30 min) to determine the stability values. Emulsion stability was calculated as:

$$\mathrm{Emulsion}\;\mathrm{stability}\;(\mathrm{EC})=\frac{V_{30}}{V_{\mathrm{el}}}\times 100$$

where: V—total volume of tube contents, V_el_—volume of the emulsified layer, V_30_—volume of the emulsified layer after heating.

#### 2.8.3. Solubility 

The solubility of the insect flours and protein concentrates was determined using a modified version of the method by Hall et al. [24]. Each sample (400 mg) was dispersed in 20 mL phosphate buffers of pH 2–10, respectively. Each buffer mixture was stirred with a magnetic stirrer bar at room temperature for 30 min and centrifuged at 7500× *g* for 20 min at 4 °C. The protein content of the supernatant and total protein in the sample was determined using the bicinchoninic acid protein assay (BCA) method with bovine serum albumin as a standard, following the manufacturer’s protocol (Sigma, St. Louis, MO, USA). Protein solubility was expressed as a percentage and calculated as follows:

$$\mathrm{Solubility}\;\left(\mathrm{ES}\right)=\frac{\mathrm{protein}\;\mathrm{content}\;\mathrm{in}\;\mathrm{supernatant}}{\mathrm{total}\;\mathrm{protein}\;\mathrm{content}\;\mathrm{in}\;\mathrm{sample}}\times 100$$

#### 2.8.4. Foaming Capacity and Stability

The foaming properties of the edible insects were measured according to Mshayisa and Van Wyk [16] with slight modifications. The edible insect and protein concentrates were rehydrated in Milli-Q water (5% *w*/*v*) followed by centrifugation at 10,000 rpm for 15 min. Then 20 mL of the supernatant was homogenised in a high shear homogeniser mixer (Polytron PT 2500E) at a speed of 16,000 rpm for three minutes. The homogenised solution was poured into a 50 mL graduated cylinder. The total volume was read at time zero and 30 min after homogenisation. The foaming capacity and foam stability were calculated from the formula:

$$\mathrm{Foaming}\;\mathrm{Capacity}\;\left(\mathrm{FC}\right)=\frac{V_{a}-V}{V}\times 100$$

$$\mathrm{Foam}\;\mathrm{Stability}\;\left(\mathrm{FS}\right)=\frac{V_{30}}{V_{a}}\times 100$$

where: V—volume before whipping (mL), V_a_—volume after whipping (mL), V_30_—volume after standing (mL). 

### 2.9. Surface Charge (Zeta Potential)

The ζ-Potential of the protein concentrates (BSFL-PC1 and BSFL-PC2) were determined using a Zetasizer Nano Series (Malvern Instruments, Malvern, Worcestershire, UK) as described by Ladjal-Ettoumi et al. [25] and Mshayisa et al. [26], respectively. The protein powders were diluted to 1% (*w*/*v*) with Milli-Q water. The Smoluchowski model was used to process data collected over least five of the experiments [27].

### 2.10. Scanning Electron Microscopy (SEM)

Scanning electron microscopy (TM-3000, Hitachi Corporation, Tokyo, Japan) was used to study the surface morphology of the BSFL flours and protein concentrates. The edible insect powders were placed onto double-sided carbon adhesive tape attached to the specimen stubs, respectively. The sample’s surface structure was observed at 320× magnification and in the secondary electron mode at 15.0 kV following the procedure described by Mshayisa et al. [26].

### 2.11. Fourier Transform Infrared Spectroscopy (FT-IR)

All flour and protein concentrate samples were analysed using a Perkin Elmer Fourier transform infrared spectroscope (FT-IR) equipped with a universal attenuated total reflectance (UATR) polarisation accessory for spectra as described by Mshayisa et al. [26]. A background spectrum was collected prior to data collection of each sample, and then the sample powders obtained by grinding in a mortar were placed directly, covering the surface of the ATR crystal. All spectra were acquired by the co-addition of 32 scans at a resolution of 4 cm^−1^ in the range of 400–4000 cm^−1^. Acetone was used to clean the UATR crystal to remove any residual contribution from previous samples. 

### 2.12. Data Analysis

All statistical tests were performed using multivariate analysis of variance (MANOVA), the level of significance was *p* < 0.05, followed by Duncan’s multiple comparisons using the statistical package for social sciences (SPSS 27, SPSS, Inc., Chicago, IL, USA).

## 3. Results and Discussion

### 3.1. Nutritional Properties

The proximate compositions of freeze-dried BSFL (BSFL-FD), defatted BSFL (BSFL-DF), alkali and isoelectric precipitation BSFL protein concentrate (BSFL-PC1), and alkaline extraction protein concentrate (BSFL-PC2) are depicted in Table 1. The ash content of all samples ranged between 2.08 and 10.81%, and BSFL-FD had the highest (*p* < 0.05) content. BSFL-PC1 had the highest protein (73.35%) content, and BSFL-FD (44.47%) had the lowest protein content (*p* < 0.05), while BSFL-PC1 had a significantly (*p* < 0.05) higher protein content compared to BSFL-PC2. This signifies that the protein extraction method affected the protein content. The protein content obtained for BSFL-FD was within a similar range to that reported by Huang et al. [28] for oven-dried BSFL (40–44%). The protein contents measured in this study were much higher than those reported by Bußler et al. [20] for fresh and freeze-fried BSFL, namely, 31.7% and 34.7%, respectively. The differences in protein content can be attributed to the different feeding regimes (diets), age, and size of the black soldier fly larvae prior to analysis. Moreover, in this study, the conversion factor of 5.60, as advised by Janssen et al. [18], was used instead of 6.25, which has been reported to overestimate the protein content since edible insects contain chitin which contributes to the nitrogen content. The protein and lipid composition of BSFL is highly impacted by what they consume [29]. Edible insects reported in this study were fed on clean, standardised feed and were fasted and blanched prior to further processing. The protein content of all the fractions investigated in this study was higher than that of common food products such as cow’s milk (3.5%), eggs (13%), fish (18.3%), and chicken (22%) [13,30]. 

As shown in Table 1, the defatting step significantly (*p* < 0.05) decreased the crude fat present in BSFL from 22.60 to 0.83%, while protein concentrates contained 0.27–0.37% fat. The fat content of BSFL-FD (22.60%) was comparable to the data reported by Bußler et al. [20] for *H. illucens* (21.1%) and *T. molitor* (20.0%). The current practice in the food industry is geared towards the extensive use of soy protein products as non-meat proteins in processed meat products. These are classified based on their protein concentration as soy flour (50–54%), concentrated soy protein (62–69%), and isolated protein (86–87%) [7]. The results of this study clearly demonstrate that BSFL flours and protein concentrates were comparable to that of soy flour and soy concentrated protein, both of which are widely used as food ingredients commercially.

### 3.2. Bulk Density and Colour

The bulk density of BSFL flour and protein concentrates is shown in Table 2. Bulk density can be described as the weight of powder per unit volume (expressed as g/mL). The bulk density of BSFL flours and protein concentrates ranged from 0.83 to1.04 g/mL. No significant differences were observed between the freeze-dried and defatted BSFL flours (*p* > 0.05). Akpossan et al. [14] reported similar bulk density values in full-fat and defatted ground *I. oyemensis* flours. In terms of the protein concentrates, a significant (*p* < 0.05) reduction in bulk density was observed compared to the flour fractions, signifying that the extraction process employed affected bulk density. In assessing packaging requirements, material handling, and applications in wet manufacturing, bulk density is a critical parameter for consideration by food processors [31]. 

Processing of the BSFL affected the visual appearance of the BSFL flour and protein concentrates produced. The colour changes are summarised in Table 2. Defatting with the hexane:isopropanol mixture led to a significantly higher (*p* < 0.05) lightness (L) in the BSF-DF than the freeze-dried BSFL flour. Similar results were reported by Mishyna et al. [32] in *A. mellifera* defatted with hexane. In this study, the defatting process significantly increased lightness (*p* < 0.05), redness (*p* < 0.05), and yellowness (*p* < 0.05). In terms of a* (red/green), only BSFL-PC2 fell within the greener quadrant (negative quadrant), while no significant (*p* > 0.05) differences were observed in the yellowness b* (positive quadrant) of all the flour and protein concentrate samples. In this study, the ΔE was in the range 11.95–23.87, indicating perceptible colour differences at a glance; in other words, the colour changes could be clearly perceived without closer inspection. The high ΔE value for BSFL-PC2 can partly be attributed to phenolic compounds situated in the insect cuticle or integument that can undergo oxidation, protein–polyphenol interaction, and enzymatic browning catalysed by phenoloxidase [7,33]. In a study conducted by Janssen et al. [33], *H. illucens* exhibited a distinct black appearance after grinding, while *T. molitor* exhibited a more deep brown colour. Therefore, the bright colour of the defatted BSFL flours could be due to the removal of compounds such as phenoloxidase, which catalyses the browning of insect flours. However, the lightness, as well as the redness, decreased significantly after further extraction. The reaction mechanism resulting in the extracted protein fractions’ dark and brown colour is still not yet well understood. Nonetheless, edible insect flours can also be used in baked goods where colour or visual appeal may not be a critical problem. 

### 3.3. Amino Acid Composition

The amino acid composition of BSFL flours and protein concentrates compared to that of cow’s milk, egg, and FAO protein intake recommendations for adults are shown in Table 3. The essential amino acids, leucine, and lysine contents were predominant in BSFL-DF compared with BSFL-FD. Lysine is considered a limiting amino acid in staple cereals such as maize, cassava, rice, and wheat [12,29]. Thus, incorporating BSFL-DF (6.76% lysine) and BSFL-PC1 (9.16% lysine) in food products can serve as a source of lysine, especially in developing countries. Histidine, an essential amino acid regarded as vital for infants and toddlers, was significantly higher (*p* < 0.05) in BSFL-DF (3.84%) and BSFL-PC1 (3.64%) than that in cow’s milk and egg (2.70% and 2.44%, respectively). The sum of the essential amino acids increased due to defatting from 24.98% to 38.20%. Moreover, BSFL-PC1 had a significantly (*p* < 0.05) higher sum of amino acids than BSFL-PC2. These values align with those of Leni et al. [34] and Huang et al. [28]. Among the non-essential amino acids in Table 3, glutamic acid had the highest concentration in BSFL-DF (13.2%), BSFL-PC1 (12.4%), and BSFL-PC2 (12.13%), respectively. These results are in agreement with the work conducted by Köhler et al. [35], who reported high levels of glutamic acid in whole house cricket (*Acheta domesticus*) flour. In this study, tryptophan was not detectable, possibly due to the fact that this amino acid is destroyed during acid hydrolysis or it was not present in the samples under investigation. The content of essential amino acids for BSFL flours and protein concentrates was comparable or exceeded FAO’s recommended amount [36] as a basic human dietary requirement. In the case of BSFL-PC1, the sum of the essential amino acids for BSFL was more than double (45.52%) the FAO requirements. The results of this study confirm the claims of other studies that, in general, edible insects are good sources of amino acids (Table 3), proteins, and lipids (Table 1) [13,35,37,38,39,40]. The high levels of essential amino acids are particularly pertinent as they cannot be synthesised by the human body in sufficient quantities and thus should be provided by the diet. Moreover, protein functionality and bioavailability are governed by the amino acid composition and the amino acid sequence. Table 4 shows the amino acid scores of BSFL Flours and protein concentrates. Leucine was the first limiting amino acid in all the samples with the exception of BSFL-FD. In terms of the BSFL protein concentrates, lysine and valine were the second limiting amino acids for BSFL-PC1 and BSFL-PC2, respectively. The essential amino acid scores for BSFL flours and protein met FAO/WHO [41] standards for older children, adolescents, and adults.

### 3.4. Techno-Functional Properties

#### 3.4.1. Water and Oil Binding Capacity

**Figure 3:**
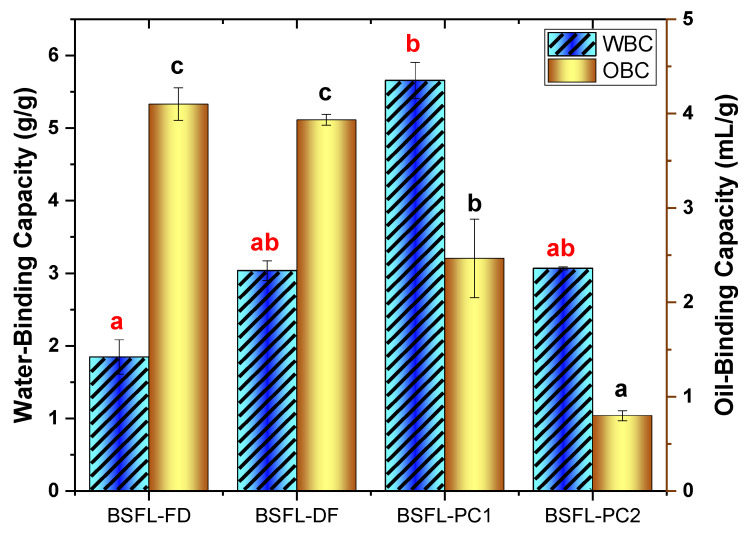
Water and oil binding capacity of flour and protein fractions. Freeze-dried BSFL flour—BSFL-FD, defatted BSFL flour—BSFL-DF, alkaline and acid precipitation extraction BSFL protein concentrate—BSFL-PC1, alkaline extraction BSFL protein concentrate—BSFL-PC2. Values are mean ± standard deviation; means with different superscript are significantly different (*p* < 0.05).

Owing to their effects on the flavour and the textural properties of food products, water and oil interactions with proteins play a significant role in food systems [12]. Protein ingredients’ water-binding capacity (WBC) and oil-binding capacity (OBC) may be influenced by intrinsic factors such as the amino acid sequence, protein conformation, hydrophobicity, and polarity. The ability of protein ingredients to interact with water under restricted conditions is expressed by its WBC. The WBCs of BSFL flour and protein fractions are displayed in Figure 3. The removal of the fat did not have a significant effect (*p* < 0.05) on the WBC of BSFL flours, whereas the BSFL-PC1 showed a higher (*p* > 0.05) WBC compared to BSFL-PC2. The differences in WBC between the protein fractions can be attributed to the differences in amino acids reported in Table 2 as influenced by the extraction method. Few studies have been conducted on the WBCs of BSFL flours and protein fractions extracted using different chemical techniques. The results of this study were higher than the WBC values described for BSFL flour fractions (0.4–0.8 g/g-) by Bußler et al. [20]. The differences in the WBC values of BSFL flours and protein concentrates can be ascribed to the differences in the methods of extraction used by the authors and the insect origin and diet. Among the BSFL flour and protein fractions, the highest WBC was observed in BSFL-PC1 (5.6 g/g). This value was comparatively higher than the WBC of other insect protein fractions reported in the literature, such as *T. molitor* (1.87 g/g) [30] and *S. gregaria* (2.18 g/g) [23]. A high water-binding capacity value for a protein allows the moistness mouthfeel and freshness of baked goods to be maintained and is correlated with a decreased moisture loss in bakery products. Thus, information about the WBC of insect-derived ingredients is essential for future application in food systems.

**Figure 4:**
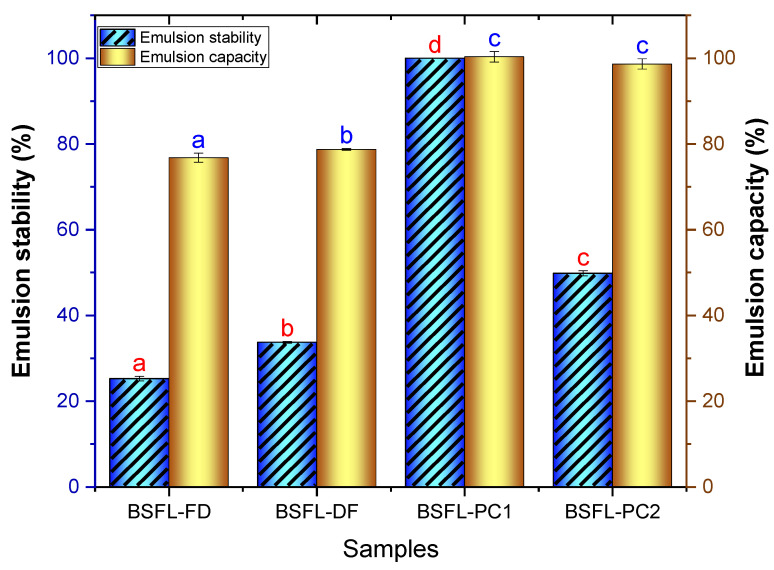
Emulsification capacity and stability of BSFL flour fractions and protein concentrates. Freeze-dried BSFL flour—BSFL-FD, defatted BSFL flour—BSFL-DF, alkaline and acid precipitation extraction BSFL protein concentrate—BSFL-PC1, alkaline extraction BSFL protein concentrate—BSFL-PC2. Values are mean ± standard deviation; means with different superscript are significantly different (*p* < 0.05).

Another primary techno-functional attribute of food ingredients used in processed foods is the OBC. To enhance the palatability and flavour retention of foods, a high OBC is desirable [12]. Figure 3 depicts the result of the OBC. Similar to the WBC, the defatting step did not have a significant (*p* > 0.05) effect on the OBC of BSFL flours, but for the BSFL protein concentrates, it was significantly lower (*p* < 0.05). The OBC of BSFL-FD flour in this study was (4.1 mL/g). This was superior to the OBC value obtained for hydrolysed migratory locust protein flour (1.5 mL/g) [21] and *T. molitor* (1.71 mL/g) [23]. The capacity of a protein to bind oil or fat is critical in the formulation of meat substitutes and extenders, as well as cake batters, sausages, and emulsions [42]. In terms of the protein fractions, the highest (*p* < 0.05) OBC was observed in BSFL-PC1 compared to BSFL-PC2 (Figure 4). The differences in OBC are possibly due to the different surface hydrophobicity or hydrophilicity of these proteins, which may be influenced by the extraction method [43]. In this research, the WBC and OBC values showed that BSFL flour and protein concentrates could be useful for various food applications, such as enhancing the palatability and texture of formulated foods.

#### 3.4.2. Emulsifying Capacity (EC) and Stability (ES)

The amphiphilic nature of proteins allows them to form and stabilise food emulsions. In the development of formulated foods, emulsifying properties are among the most vital properties, and strong emulsifying properties are needed to produce meat analogues and milk-like drinks [44]. Figure 4 exhibits the emulsion capacity and stability of the BSFL flour and protein concentrates. The emulsion capacity of defatted BSFL flour (BSFL-DF, 78.73%) was significantly higher (*p* < 0.05) than the full-fat flour (BSFL-FD, 76.80%). Similar results were reported for the EC of I. oyemensis by Akpossan et al. [14], while Kim et al. [8] observed a lower EC of cricket flour (39.17–45%). In a study conducted by Mishyna et al. [32], no significant differences were observed in raw and defatted S. gregaria flours. The variations in the EC of edible insect flours could be attributed to protein content and molecular structure differences. In general, the EC of a food protein is based on the protein–oil and protein–water interactions. The highest EC was determined for the protein concentrates BSFL-PC1 (100%) and BSFL-PC2 (100%). These results show that the processes used to obtain protein concentrates did not have a significant (*p* > 0.05) impact on the ability of BSFL-PC1 and BSFL-PC2 to aid in the creation of emulsions. 

In terms of ES, BSFL-FD (25.27%) formed emulsions with a significantly (*p* < 0.05) lower ES compared with BSFL-DF (33.73%), signifying that its protein did not effectively interact at the interface to form a strong interfacial membrane (Figure 4). The ES of the protein concentrates (49.83–100%) was significantly higher (*p* < 0.05) than the flour fractions (25.27–33.73%). The BSFL protein concentrate results reported in this study indicate that these novel proteins may be appropriate for use in the formulation of a wide range of processed food products. Currently, there is a paucity of studies investigating the emulsification properties of insect protein concentrates obtained using different extraction techniques (alkaline and acid precipitation and alkaline extraction), which makes the comparison of the results difficult. Proteins currently used by the food industry due to their emulsifying abilities are mainly derived from soybean, milk (whey or casein), and egg. These are widely used in various food formulations due to their commercial availability and good functional properties [45]. However, the major drawback is that they all have been identified as common food allergens. Therefore, further studies on the emulsifying properties of edible insect concentrates are required, especially using different extraction methods. Based on the findings of this study, it could be concluded that BSFL flours and protein concentrates possess emulsifying properties. 

#### 3.4.3. Protein Solubility

In order to provide knowledge on the successful use of insect-derived ingredients in different food applications, the protein solubility of edible insect flours and protein concentrates was investigated. Solubility at different pH values and the level of protein denaturation due to heat or chemical treatment serve as a measure of how well insect flours and protein concentrates can perform when integrated into food systems [46]. 

**Figure 5:**
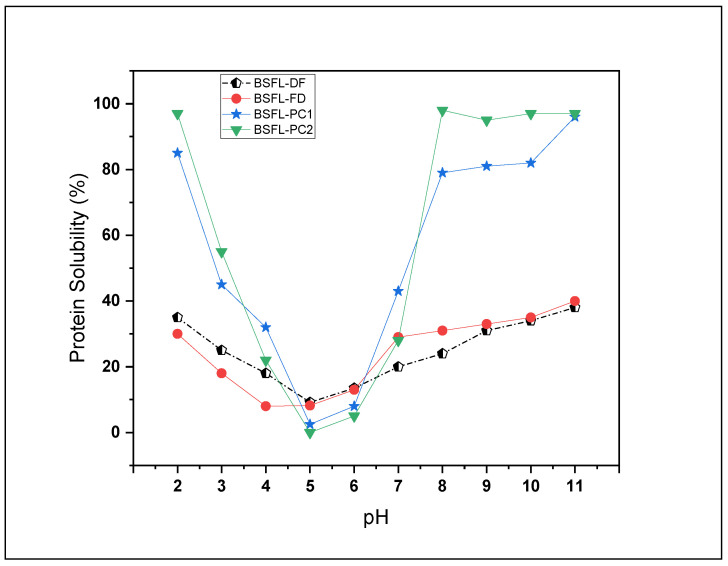
Solubility profile of BSFL flour fractions and protein concentrates as a function of pH. Freeze-dried BSFL flour (pentagon), defatted BSFL flour (circle), alkaline and acid precipitation extraction BSFL protein concentrate (star), and alkaline extraction BSFL protein concentrate (triangle).

In general, BSFL-FD and BSFL-DF flours had a low protein solubility at pH 2–3 compared to the protein concentrates (Figure 5). The high solubility at low pH values for the protein concentrates (BSFL-PC1, 95% and BSFL-PC2, 85%) makes them ideal candidates for use in acidic beverages. The protein solubility of all BSFL samples ranged from a minimum at the isoelectric point (pI) to its maximum at pH 11 (Figure 5). For all the insect flours, the pI was in the region of pH 4.0–4.5. During the extraction process, protein solubility was highly dependent on the pH. The results of this study resemble the pI of common essential food proteins such as casein (4.6), soybean (4.5), and meat products (5.0) [12,13,46]. These results can be attributed to the reduced interaction between protein and water at pH 4.5–5.0, and this phenomenon enhances the protein–protein interaction in foods resulting in protein aggregation and precipitation [46]. The protein solubility profiles of BSFL flours and protein concentrates against pH were generally similar to each other and consistent with previously published data for insect species [14,24,47] and plant legumes such as peas, Kabuli chickpeas, and kidney beans [12,31]. Protein concentrates must be highly soluble in order to be used as functional ingredients in a wide variety of foods, including confections, salad dressings, coffee whiteners, whipped toppings, and beverages [12,32]. These findings further support the possible application of BSFL flour, and BSFL protein concentrates over a broad pH spectrum, in addition to acidic foods. 

#### 3.4.4. Foaming Capacity and Foam Stability

**Figure 6:**
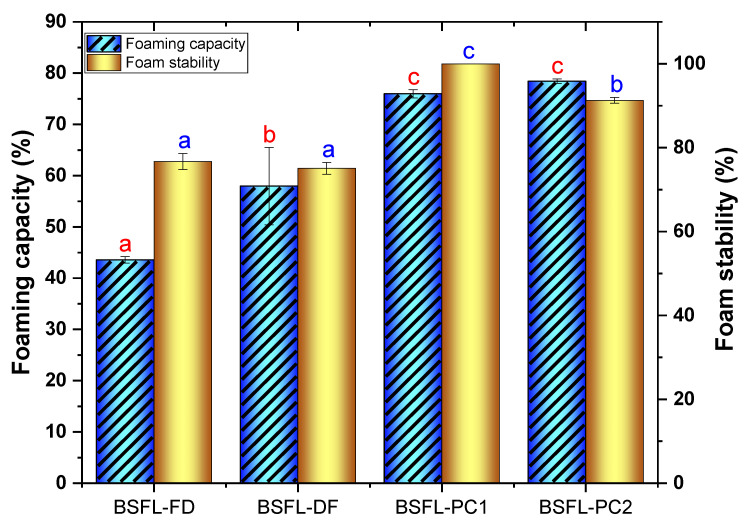
Foaming capacity and stability of BSFL flour and protein concentrates. Freeze-dried BSFL flour—BSFL-FD, defatted BSFL flour—BSFL-DF, alkaline and acid precipitation extraction BSFL protein concentrate—BSFL-PC1, alkaline extraction BSFL protein concentrate—BSFL-PC2. Values are mean ± standard deviation; means with different superscript are significantly different (*p* < 0.05).

Food foams can be defined as air bubbles imprisoned in a liquid and stabilised by protein at the air–liquid interface. As foaming agents, proteins play a significant role in the distribution of fine air cells in the processed food structure. They are also responsible for imparting smoothness and lightness and allowing flavours to be volatilised to improve the palatability of food products [12]. The foam capacity and stability results for BSFL flour and protein concentrates are exhibited in Figure 6. The FC of BSFL-FD (40%) was not statistically different (*p* > 0.05) compared with that of BSFL-DF (55%). The foaming capacity of BSFL-PC2 (78.43%) after alkaline extraction was significantly higher (*p* < 0.05) than BSFL-PC1 (75.97%). Overall, the protein concentrates exhibited an improved foaming capacity. The high foamability may be attributed to increased protein content and the possibility of changes in protein characteristics after the extraction. The FC results of this study show that BSFL-FD and BSFL-DF flours were higher than those described by Adebowale et al. [37] for the large African cricket (*Gryllidae* sp.) that had an FC of only 6%. Previous studies on freeze-dried *A. mellifera* and *S. gregaria* conducted by Mishyna et al. [32] also exhibited low FC of 5.8% and 45%, respectively. These results are consistent with the observation of Akpossan et al. [14], who reported a poor FC of the edible full-fat insect *I. oyemensis* flour. The variations in FC may be due to the different conformation characteristics of edible insect proteins.

The stabilisation of foams is primarily dependent on the formation of a thick cohesive viscoelastic film involving each gas bubble. The present study supported earlier findings that foam stability increases with the removal of fat. To our best knowledge, this is the first study to investigate the effect of protein extraction methods on the FC and FS of BSFL protein concentrates. This work will generate fresh insights into the effect of extraction methods on edible insect protein techno-functional properties. An increased FC and FS of protein fractions is also consistent with higher protein solubility (Figure 5) compared to BSFL flours, which likely contributes to the formation of a cohesive viscoelastic film at the interface via intermolecular interactions. The high FC and FS of BSFL-PC1 and BSFL-PC2 reported in this study suggest that they can be used in bakery and confectionery products. Currently, the food industry utilises wheat, soy, and dairy-based protein concentrates and isolates as ingredients. However, consumers and food processors are searching for new novel protein sources to alleviate the allergenicity challenges posed by the common eight priority allergens (wheat, peanut, soy, fish, dairy, tree nuts, crustaceans, and egg). 

#### 3.4.5. Effect of pH on the ζ-Potential of BSFL Protein Concentrates

**Figure 7:**
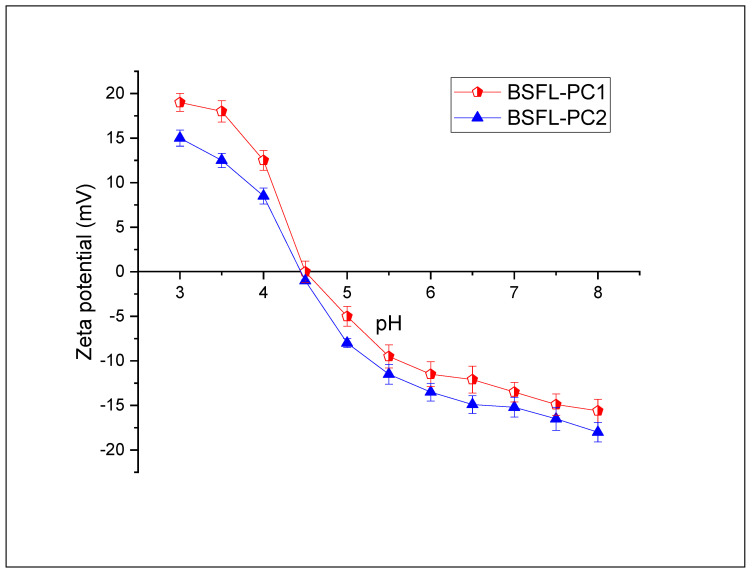
Effect of pH on the zeta potential of BSFL protein concentrates. The point where the line crosses the x-axis represents the apparent pI of the protein solution (prepared in MilliQ water).

A significant feature of proteins which determines their functional properties is the surface charge. In most cases, protein molecules carry a charge, which plays an essential role in interacting with other food matrices components [48]. Protein Zeta potential (ζ) is a critical analysis tool that can be used to optimize food product formulations for new ingredients, predict interactions with surfaces, and predict long-term stability. Zeta (ζ) potential measurements of protein dispersions for different pH values provide information about the isoelectric point (pI). The ζ-potential of BSFL protein concentrates is displayed in Figure 7. The apparent isoelectric point (pI) of all proteins together was determined at a zero net charge. The pH-dependent droplet ζ-potential of BSFL proteins extracted with both methods was zero at pH 4.5, representing the pI of the isolates at this pH. Form the results, it is clear that the chemical extraction method does not affect the isoelectric point of the BSFL protein concentrates. Zeta potential values (positive or negative) at the curve’s extremes typically indicate increased electrostatic repulsion, increasing protein solubility. Protein solubility around the apparent pI, on the other hand, was the lowest since there was slight repulsion. These results are in agreement with previously reported data for BSFL [49]. Typically, proteins (e.g., from milk, soy, egg) used as ingredients in the food industry have an isoelectric point in the pH range 4–6 [45]. Therefore, the respective droplet ζ-potentials converged to zero in this pH range to finally change from positive to negative values at pH values above the pI. The pI tended to coincide with the minimum solubility, as previously shown in Figure 3.

### 3.5. Scanning Electron Microscopy (SEM)

**Figure 8:**
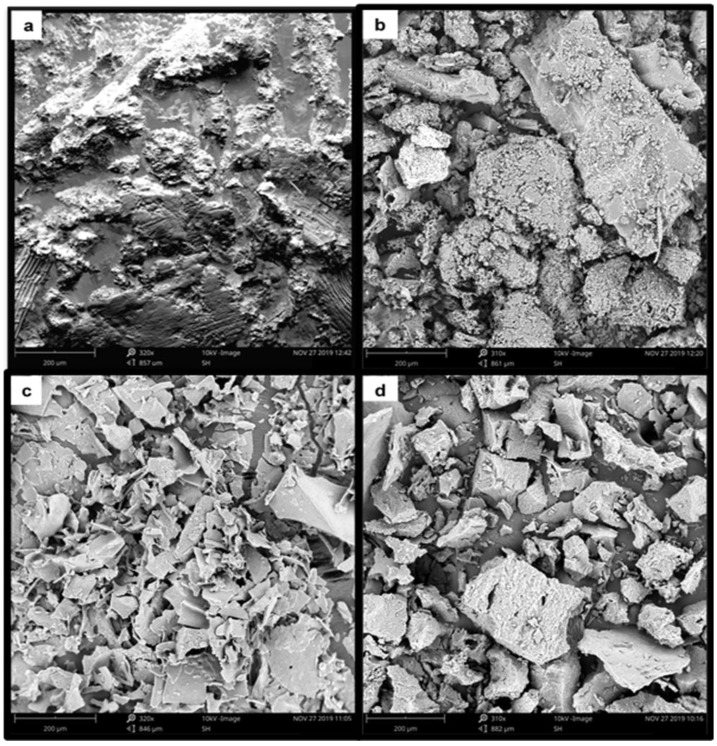
Scanning electron micrographs of (**a**) BSFL-FD flour, (**b**) BSFL-DF flour, (**c**) BSFL-PC1, and (**d**) BSFL-PC2.

The study of the microstructure of BSFL flours and protein concentrates provided further information on the results of the physicochemical and functional parameters and allowed a more complete interpretation of the effects produced by the different treatments. The surface morphology and microstructure of BSFL flours and protein concentrates are shown in Figure 8. The BSFL freeze-dried and defatted flours showed particle morphology and distribution differences, with BSFL-DF exhibiting large particles. The microstructure of BSFL-FD flour was less dense and had a smooth surface, although some irregular, cracked, or shrunk particles could also be observed, while the BSFL-DF was largely irregularly shaped. The BSFL-PC1 microstructure exhibited a thin flaky plate-like surface morphology, whereas the surface morphology of BSFL-PC2 appeared large and blocky. There were distinct differences in the microstructure of the protein concentrates. These findings suggest that the protein extraction methods changed or modified the microstructure of the BSFL proteins and further explain the observed differences in the functional and physicochemical properties. Moreover, more work on higher resolution or magnification should be further conducted with the view to characterise the BSFL flours and protein concentrates fully.

### 3.6. Fourier Transform Infrared (FT-IR) Spectrometer Analysis

**Figure 9:**
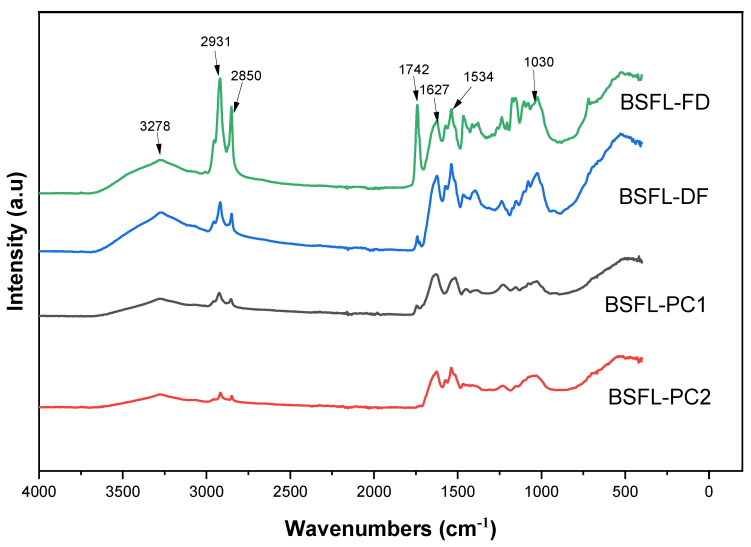
FT-IR Spectra of BSFL flours and protein concentrates. Freeze-dried BSFL flour—BSFL-FD, defatted BSFL flour—BSFL-DF, alkaline and acid precipitation extraction BSFL protein concentrate—BSFL-PC1, alkaline extraction BSFL protein concentrate—BSFL-PC2.

FT-IR, a precise, low-cost, and non-destructive analytical technique, was employed to examine the effect of the protein extraction technique on the protein secondary structure (functional groups). Figure 9 presents representative FT-IR absorption spectra for BSFL flours and protein concentrates in the 400–4000 cm^−1^ region. The major peaks for BSFL samples in this study were found at wavenumbers 3278, 2931, 2580, 1742, 1627, and 1534 cm^−1^ for amide A, amide B, amide I, amide II, and amide III, respectively. The absorption peaks of all samples at 2931 and 2850 cm^−1^ represent the functional groups O-H and C-H, respectively. After defatting and protein extraction, the intensity of these peaks decreased significantly. This can be attributed to the chemical treatment applied. Vital information on the protein secondary structure is provided by the Amide I band (1650–1800 cm^−1^) resulting from the stretching of the C=O of amide in protein. Its intensity decreased as a function of protein extraction and thus indicated an alteration of the protein structure. The Amide I and II bands are the most important. The sensitivity of C=O peptide bonds to the different conformations of protein secondary structures is mainly due to these bands. The protein concentrate (BSFL-PC1 and BSFL-PC2) samples showed a significantly lower peak intensity at 1627 cm^−1^ according to the FT-IR spectrum presented in Figure 9, which corresponds to native intramolecular β-sheets and had marginally lower α-helices intensities (1652 cm^−1^) compared to the BSFL-FD sample [50,51]. The IR spectra of BSFL-FD and BSFL-DF were composed of the distinct regions that correspond to the amide I band (1600–1700 cm^−1^), which is mostly carbonyl stretching (C=O) vibrations and amide II which is essentially the combination of the N-H plane boundary and C-N stretching vibrations. It is widely acscepted that the functional and digestive properties of proteins are directly related to their molecular structure, wherein the configurations of the α-helix and beta-sheet are associated with their performance in food systems, particularly their absorption of water and digestion in vitro [50,51]. The BSFL flour and protein concentrate FT-IR spectra were largely comparable. However, variations were observed in a few characteristic peaks and intensities (Figure 9), suggesting minor differences in structure, amino acids, and protein function groups. In addition, the prolonged exposure of proteins during freeze-drying to low temperatures may have caused an increase in disordered structures. Thus, the extraction process influences the secondary structure of the proteins.

## 4. Conclusions

This study established the nutritional properties of BSFL flour fractions and protein concentrates. All fractions met the recommended FAO requirements for a well-balanced essential and non-essential amino acid content for human consumption. The overall results indicated that alkaline and acid precipitation extractions of BSFL protein concentrates resulted in enhanced nutritional and functional properties. The protein extraction method appeared to have altered the molecular structure and characteristics of proteins such as surface charge and functional groups and thus contributed to the improved functionality of protein fractions. As a general trend, an improvement in water binding capacity, solubility, and emulsifying capacity were observed. Protein concentrates (BSFL-PC1 and BSFL-PC2) extracted from BSFL exhibited a high emulsion capacity. This offers the possibility of the industrial processing of these edible insect protein concentrates and their use in suitable commercial food applications. More research on the interaction of edible insect ingredients with other food components and on the microbiological, rheological, and sensory properties of new insect-based food proteins is recommended for future studies.

## Figures and Tables

**Table 1:** Proximate composition of BSFL flour and protein concentrates.

Sample	Crude Protein (%)	Crude Fat (%)	Carbohydrates (%)	Moisture (%)	Ash (%)
BSFL-FD	44.47 ± 1.77 ^a^	22.60 ± 1.39 ^b^	21.13 ± 0.57 ^a^	9.48 ± 0.34 ^c^	10.81 ± 0.26 ^d^
BSFL-DF	50.12 ± 0.66 ^b^	0.83 ± 0.06 ^a^	40.80 ± 0.39 ^d^	6.07 ± 0.25 ^b^	8.25 ± 0.33 ^c^
BSFL-PC1	73.35 ± 0.88 ^d^	0.37 ± 0.12 ^a^	22.92 ± 1.02 ^b^	1.48 ± 0.01 ^a^	3.36 ±0.28 ^b^
BSFL-PC2	68.47 ± 0.93 ^c^	0.27 ± 0.06 ^a^	29.19 ± 0.92 ^c^	1.57 ± 0.03 ^a^	2.08 ±0.01 ^a^

Freeze-dried BSFL flour—BSFL-FD, defatted BSFL flour—BSFL-DF, alkaline and acid precipitation extraction BSFL protein concentrate—BSFL-PC1, alkaline extraction BSFL protein concentrate—BSFL-PC2. Results are reported as mean ± standard deviation. Different superscripts in the column indicate significant differences between treatments (*p* ≤ 0.05).

**Table 2:** Bulk density and colour attributes of black soldier fly larvae flours and protein concentrates.

Sample	Bulk Density (g/mL)	L *	a *	b *	ΔE
BSFL-FD	1.01 ± 0.02 ^b^	51.24 ± 0.34 ^c^	0.84 ± 1.34 ^ab^	17.99 ± 1.70 ^a^	Control
BSFL-DF	1.04 ± 0.02 ^b^	62.95 ± 1.01 ^d^	1.67 ± 0.12 ^b^	18.43 ± 2.67 ^a^	11.95 ± 1.16 ^a^
BSFL-PC1	0.84 ± 0.01 ^a^	44.56 ± 0.63 ^b^	0.85 ± 1.35 ^ab^	17.99 ± 1.70 ^a^	16.16 ± 1.27 ^a^
BSFL-PC2	0.86 ± 0.01 ^a^	35.20 ± 1.87 ^a^	−2.14 ± 3.28 ^a^	18.78 ± 6.56 ^a^	23.87 ± 4.16 ^b^

Freeze-dried BSFL flour—BSFL-FD, defatted BSFL flour—BSFL-DF, alkaline and acid precipitation extraction BSFL protein concentrate—BSFL-PC1, alkaline extraction BSFL protein concentrate—BSFL-PC2. Results are reported as mean ± standard deviation. Different superscripts in the column indicate significant differences between treatments (*p* ≤ 0.05).

**Table 3:** Amino acid composition (g.100 g^−1^) of BSFL flour fractions and protein concentrates compared to cow’s milk, egg protein, and FAO requirements for human consumption.

Amino Acid	BSFL-FD	BSFL-DF	BSFL-PC1	BSFL-PC2	Cow’s Milk	Egg Protein	FAO [36]
Essential							
Histidine	1.82 ± 0.01 ^a^	3.84 ± 0.01 ^d^	3.64 ± 0.02 ^c^	2.48 ± 0.00 ^b^	2.70	2.40	1.50
Isoleucine	2.49 ± 0.01 ^a^	4.32 ± 0.44 ^bc^	5.18 ± 0.78 ^c^	3.99 ± 0.45 ^b^	4.90	5.60	3.00
Leucine	4.01 ± 0.12 ^a^	7.29 ± 0.15 ^c^	7.99 ± 0.29 ^d^	4.61 ± 0.17 ^b^	9.10	8.30	5.90
Lysine	3.73 ± 0.01 ^a^	6.79 ± 0.19 ^c^	9.16 ± 0.34 ^d^	5.37 ± 0.20 ^b^	7.40	6.30	4.5
Methionine	2.53 ± 0.00 ^a^	1.72 ± 0.45 ^a^	2.53 ± 0.00 ^a^	2.67 ± 0.08 ^a^	2.60	3.20	1.60
Phenylalanine	4.14 ± 0.01 ^a^	4.36 ± 0.20 ^a^	7.18 ± 0.05 ^c^	5.41 ± 0.21 ^b^	4.90	5.10	Not supplied
Threonine	2.87 ± 0.53 ^a^	4.09 ± 0.49 ^b^	4.95 ± 0.12 ^bc^	4.65 ± 0.00 ^b^	4.40	5.10	2.30
Valine	3.39 ± 0.01 ^a^	5.80 ± 0.51 ^b^	5.61 ± 0.89 ^c^	5.09 ± 0.51 ^b^	6.60	7.60	3.90
**Sum**	**24.98 ± 0.80 ^a^**	**38.20 ± 2.44 ^b^**	**46.52 ± 2.48 ^c^**	**34.27 ± 2.19 ^b^**	**42.60**	**43.60**	**22.70**
Non-essential							
Alanine	3.58 ± 0.01 ^a^	5.95 ± 0.48 ^c^	4.66 ± 0.04 ^b^	5.41 ± 0.48 ^bc^	3.60	5.40	-
Arginine	3.38 ± 0.00 ^a^	4.85 ± 0.36 ^b^	4.57 ± 0.02 ^b^	5.72 ± 0.02 ^c^	3.60	6.10	-
Aspartic acid	5.89 ± 0.01 ^a^	10.38 ± 0.39 ^b^	12.56 ± 0.69 ^c^	9.62 ± 0.40 ^a^	7.70	10.70	-
Glycine	3.08 ± 0.06 ^a^	4.80 ± 0.13 ^c^	3.88 ± 0.06 ^b^	4.97 ± 0.15 ^c^	3.00	2.00	-
Glutamic acid	6.89 ± 0.05 ^a^	13.62 ± 0.05 ^d^	12.13 ± 0.09 ^b^	12.40 ± 0.05 ^c^	12.0	20.60	-
Proline	2.57 ± 0.01 ^a^	5.71 ± 0.23 ^d^	4.38 ± 0.01 ^c^	3.38 ± 0.24 ^b^	8.50	3.80	-
Serine	2.13 ± 0.04 ^a^	4.42 ±0.10 ^d^	3.99 ± 0.28 ^c^	3.13 ± 0.17 ^b^	5.20	7.90	-
Tyrosine	6.89 ± 0.00 ^c^	6.47 ± 0.07 ^b^	6.28 ± 0.15 ^a^	9.34 ± 0.09 ^b^	4.10	7.60	-
**Sum**	**34.41 ± 0.21 ^a^**	**56.21 ± 1.74 ^b^**	**52.45 ± 1.85 ^b^**	**53.98 ± 2.00 ^b^**	**55.30**	**56.50**	**-**

Freeze-dried BSFL flour—BSFL-FD, defatted BSFL flour—BSFL-DF, alkaline and acid precipitation extraction BSFL protein concentrate—BSFL-PC1, alkaline extraction BSFL protein concentrate—BSFL-PC2. Results are reported as mean ± standard deviation of triplicate analysis. Different letters indicate significant (*p* < 0.05) differences between the means across the rows.

**Table 4:** Amino acid score of BSFL flours and protein concentrates and the FAO/WHO/UNU (2007) consultation.

Amino Acids	FAO/WHO/UNU 2007 [41] (mg/g Protein)	Chemical Score (%)			
		BSFL-FD	BSFL-DF	BSFL-PC1	BSFL-PC2
Histidine	15	191.84	254.03	175.72	230.52
Isoleucine	30	139.86	152.62	150.66	174.96
Leucine	59	110.73 ^b^	126.51 ^c^	85.67 ^b^	132.72 ^b^
Lysine	45	130.74 ^c^	149.90	126.75 ^c^	193.37
Methionine	16	265.73	113.68 ^b^	188.91	160.22
Threonine	23	193.08	173.10	210.92	200.63
Valine	39	142.76	153.46	144.30	142.11 ^c^
Phenylalanine + tyrosine	38	488.83	301.99	439.71	358.91
Total EAA	277	151.8	146.1	140.2	169.1

^b^ First limiting amino acid, ^c^ Second limiting amino acid, Freeze-dried BSFL flour—BSFL-FD, defatted BSFL flour—BSFL-DF, alkaline and acid precipitation extraction BSFL protein concentrate—BSFL-PC1, alkaline extraction BSFL protein concentrate—BSFL-PC2.

## Data Availability

The datasets used and/or analyzed during the current study are available from the corresponding author on reasonable request.
